# THz Waves Improve Spatial Working Memory by Increasing the Activity of Glutamatergic Neurons in Mice

**DOI:** 10.3390/cells14050370

**Published:** 2025-03-03

**Authors:** Lequan Song, Zhiwei He, Ji Dong, Haoyu Wang, Jing Zhang, Binwei Yao, Xinping Xu, Hui Wang, Li Zhao, Ruiyun Peng

**Affiliations:** Beijing Institute of Radiation Medicine, 100850 Beijing, China

**Keywords:** terahertz waves, glutamatergic neurons, synaptic plasticity, synaptic transmission, working memory, multiomics

## Abstract

Terahertz (THz) waves, a novel type of radiation with quantum and electronic properties, have attracted increasing attention for their effects on the nervous system. Spatial working memory, a critical component of higher cognitive function, is coordinated by brain regions such as the infralimbic cortex (IL) region of the medial prefrontal cortex and the ventral cornu ammonis 1 (vCA1) of hippocampus. However, the regulatory effects of THz waves on spatial working memory and the underlying mechanisms remain unclear. In this study, the effects of 0.152 THz waves on glutamatergic neuronal activity and spatial working memory and the related mechanisms were investigated in cell, brain slice, and mouse models. Cellular experiments revealed that THz waves exposure for 60 min significantly increased the intrinsic excitability of primary hippocampal neurons, enhanced glutamatergic neuron activity, and upregulated the expression of molecules involved in glutamate metabolism. In brain slice experiments, THz waves markedly elevated neuronal activity, promoted synaptic plasticity, and increased glutamatergic synaptic transmission within the IL and vCA1 regions. Molecular dynamics simulations found that THz waves could inhibit the ion transport function of glutamate receptors. Moreover, Y-maze tests demonstrated that mice exposed to THz waves exhibited significantly improved spatial working memory. Multiomics analyses indicated that THz waves could induce changes in chromatin accessibility and increase the proportion of excitatory neurons. These findings suggested that exposure to 0.152 THz waves increased glutamatergic neuronal activity, promoted synaptic plasticity, and improved spatial working memory, potentially through modifications in chromatin accessibility and excitatory neuron proportions.

## 1. Introduction

Terahertz (THz) waves, as high-frequency electromagnetic radiation, have attracted significant attention in the biomedical field in recent years [1]. Their potential effects at the cellular, tissue, and molecular levels have been extensively studied. In the field of neuroscience, the regulatory effects of THz waves on neuronal function have become a focal point of research. While evidence indicates that THz waves can affect neuronal excitability and synaptic function, their specific regulatory effects on glutamatergic neurons and the related mechanisms remain poorly understood [2]. Glutamatergic neurons, the primary excitatory neurons in the central nervous system, are essential for various physiological processes, including learning, memory, and synaptic plasticity [3]. These neurons mediate synaptic transmission through complex signal transduction pathways and are especially critical in brain regions that are integral to working memory and spatial learning, such as the infralimbic cortex (IL) and ventral cornu ammonis 1 (vCA1) [4]. Advances in neuroscience technologies, such as patch-clamp, calcium imaging, and high-density microelectrode arrays (HD-MEA), have enabled precise studies of neuronal excitability and synaptic transmission. Omics technologies, including assays for transposase-accessible chromatin using sequencing (ATAC-seq), high-throughput chromosome conformation capture (Hi-C), and single-nucleus RNA sequencing (snRNA-seq), have provided novel insights into chromatin accessibility, gene expression, and cell type composition, further broadening our understanding of neuronal function.

Research has suggested that THz waves exposure under certain conditions has positive regulatory effects on neuronal function. A study revealed that THz waves influence ion permeability in neuronal membranes, leading to increased intracellular concentrations of Ca^2+^ and Na^+^, decreased K^+^ levels, and subsequent neuronal depolarization and excitability [5]. At the synaptic level, THz waves could increase excitatory synaptic transmission and firing activity in primary cortical neurons, and improve the synaptic transmission efficiency in hippocampal slices for more than 10 min [6]. At the behavioral level, THz waves have shown beneficial effects. In APP/PS1 transgenic mice, THz waves reduced the amyloid β (Aβ) plaque burden and improved cognitive function, as evidenced by the results of the Morris water maze and shuttle box tests [7]. Additionally, in healthy animals, THz waves alleviated anxiety and depressive behaviors and increased social interactions [8]. However, a few studies reported contradictory effects, with increased anxiety and depressive-like behaviors observed in some animal models [9]. These findings suggest that the effects of THz waves might depend on exposure conditions and experimental models, highlighting the need for further research to elucidate specific mechanisms.

This study aimed to systematically elucidate the effects of THz waves on neuronal function and spatial working memory while exploring the molecular mechanisms underlying these neuromodulatory effects. Using electrophysiology, molecular biology, molecular dynamics, and calcium imaging techniques, we investigated the impact of THz waves on neuronal excitability, synaptic plasticity, and calcium signaling. Behavioral experiments were conducted to assess spatial working memory. Furthermore, we explored epigenomic and genomic alterations induced by THz waves, focusing on chromatin accessibility, three-dimensional genome organization, and cell type composition. This work provides critical insights into the neuromodulatory effects of THz waves and establishes a foundation for their potential biomedical applications.

## 2. Methods

### 2.1. Animals

All animal experiments and procedures were approved by the Institutional Animal Care and Use Committee (IACUC-DWZX-2024-574). Conscious and unrestrained C57BL/6N male mice aged 6 weeks were used in this study. The mice were housed in a specific pathogen-free environment within a laboratory animal facility, which was maintained at temperatures between 21 °C and 23 °C and humidity levels of 40% to 60%, under a 12 h light/dark cycle. Under standard conditions, 5 mice were housed per cage, while those that underwent surgical procedures were housed individually. The mice had unrestricted access to standard rodent chow and water unless otherwise specified. All experiments were conducted during the daytime under a light/dark cycle in strict adherence to institutional guidelines.

### 2.2. Neurons Culture

Primary hippocampal neurons were prepared as described in a previously published manuscript [10]. Hippocampal neurons from postnatal day 0 (P0) Wistar rat pups were obtained by decapitation, followed by meticulous isolation of the hippocampi under a microscope. The isolated hippocampus was placed in precooled high-glucose DMEM and subsequently minced into small pieces. After digestion for 10 min, the cell suspension was centrifuged at 80× *g* for 5 min at room temperature, and the supernatant was discarded. The cell pellet was resuspended to a density of 5 × 10^5^ cells/mL in Neurobasal-A medium. The cells were plated on 35 mm culture dishes for Western blot and on glass slides for in situ hybridization and electrophysiological analyses. All the cultures were maintained in an incubator with 5% CO_2_ at 37 °C.

### 2.3. Brain Slice Preparation

Mice were anesthetized with isoflurane, followed by transcardial perfusion with 20 mL of oxygenated, ice-cold modified artificial cerebrospinal fluid (aCSF). The composition of the modified aCSF was as follows: 10 mM NaCl, 2.5 mM KCl, 1.25 mM NaH_2_PO_4_, 25 mM NaHCO_3_, 0.5 mM CaCl_2_, 7 mM MgCl_2_, 195 mM sucrose, 10 mM glucose, and 2 mM sodium pyruvate. Osmolarity was maintained at 300–305 mOsm/kg, and the pH was adjusted to 7.35–7.45 with NaOH. Coronal brain sections (300 μm thick) were prepared using a vibratome (Campden, London, UK). The brain slices were initially allowed to recover in modified aCSF at 32 °C for 30 min, followed by incubation in oxygenated standard aCSF at room temperature for a minimum of 1 h. The standard aCSF used for incubation comprised 119 mM NaCl, 2.5 mM KCl, 1.25 mM NaH_2_PO_4_, 24 mM NaHCO_3_, 2 mM CaCl_2_·2H_2_O, 2 mM MgSO_4_·7H_2_O, and 12.5 mM glucose, with the osmolarity maintained at 300–305 mOsm/kg and the pH adjusted to 7.35–7.45 with NaOH. All chemicals utilized for brain slice preparation were procured from Sigma (St. Louis, MO, USA).

### 2.4. THz Waves Exposure

THz waves were generated using a QS2-180-HP source (Microtech Instruments, Eugene, OR, USA). This quasioptical system uses a QS2-180 backwave oscillator (BWO) with a frequency range of 114–2349 GHz. In addition, for the BWO-based THz wave source, each frequency corresponds to a specific output power. The average power density at 0.152 THz was 12.6 mW/cm^2^. A schematic diagram of THz waves exposure is shown in Figure S1. For in vitro THz waves exposure, to prevent aCSF from absorbing the THz waves, they were emitted from the bottom of the chamber. A THz lens (TYDEX, Saint Petersburg, Russia) was used to focus the scattered light into a directional beam. In this study, the cells were placed in a CO_2_ incubator maintained at 37 °C. The brain slices were continuously perfused with oxygen-saturated aCSF during the exposure period. The samples were divided into two groups: the control (C) group and the terahertz (T) group. The T group was exposed to 0.152 THz for 60 min, whereas the C group was not exposed to THz waves.

For in vivo THz waves exposure, the QS2-180 THz wave source was placed horizontally on an optical platform. The THz waves were focused at the focal point after passing through a 4-inch polytetrafluoroethylene lens. One end of the THz fiber was placed at the focal position, and the other end was fixed on the surface of the mouse skull to irradiate the target brain region. For THz waves exposure in the IL, the mice underwent a protocol to make their skulls transparent, and the THz wave fiber was then placed directly above the skull for THz waves exposure. The procedure for creating a transparent skull has been described in detail in the published literature [11]. Briefly, the mice were anesthetized with isoflurane and secured in a stereotaxic apparatus. Following an incision to expose the skull, the periosteum was carefully scraped away using an ophthalmic scalpel to ensure a clean and dry skull surface. A thin layer of Krazy Glue (Loctite, Düsseldorf, Germany) was applied with a metal rod, allowed to dry, and subsequently coated with a layer of nail polish. For THz waves exposure in the vCA1, a THz ferrule with a diameter of 1 mm was implanted into the vCA1 (AP: −3.15 mm, ML: ±3.15 mm, DV: −4.15 mm). The ferrule was coupled to the optical fiber through a converter to achieve THz waves exposure in the vCA1. After two weeks, inflammation subsided, rendering the skull surface transparent and suitable for further experimentation.

### 2.5. Stereotaxic Surgery

Mice were anesthetized via isoflurane and securely positioned in a stereotaxic frame (RWD Life Science, Shenzhen, China). A small incision was made at the planned craniotomy site, and the overlying muscles were removed. A symmetrical craniotomy was performed using a microdrill equipped with a 0.5 mm ultrafine burr (RWD Life Science, Shenzhen, China). Glass micropipettes with tip diameters of 10–20 μm were prepared using a micropipette puller (Lianqi Future, Wuhan, China) for adeno-associated virus (AAV) microinjection and connected to a microinjection pump (RWD Life Science, Shenzhen, China) after being prefilled with silicone oil to prevent air infiltration. The AAVs utilized in this study included rAAV-CaMKIIa-GCaMP8M, rAAV-CaMKIIa-ChrimsonR-mCherry, rAAV-CaMKIIa-mCherry and rAAV-CaMKIIa-ArchT-EGFP. The injection coordinates were as follows: vCA1 (AP: −3.15 mm, ML: ±3.15 mm, DV: −4.15 mm) and IL (AP: +1.6 mm, ML: ±0.3 mm, DV: −2.3 mm). AAV solutions were injected into the vCA1 at a rate of less than 50 nL/min (0.15 μL per side bilaterally). After injection, the micropipette remained in place for 10 min to ensure thorough diffusion of the viral material into the target region. For the optogenetic experiments, the skulls of the mice were either cleared or implanted with ceramic fibers. The mice were allowed to recover for at least three weeks prior to exposure to THz waves and subsequent experiments.

### 2.6. Slice Electrophysiology

Patch clamping of brain slices has been previously described [12]. Patch clamp recordings were conducted using patch pipettes with an impedance of 4–6 MΩ. In addition to IPSC recordings, a KCl electrode solution containing 140 mM KCl, 4 mM MgCl_2_, 2 mM Na_2_ATP, 0.3 mM Na_2_GTP, 10 mM HEPES, and 0.1 mM EGTA was used. All other electrophysiological recordings employed a potassium gluconate electrode solution containing 130 mM K-gluconate, 5 mM NaCl, 2 mM MgCl_2_, 4 mM Na_2_ATP, 0.4 mM Na_2_GTP, 10 mM HEPES, 0.5 mM EGTA, and 10 mM Na_2_-phosphocreatine, with an osmolarity of 300–305 mOsm/kg and a pH of 7.35–7.45 for all patch pipette solutions. Prior to recording, the slices were transferred to the recording chamber and perfused with oxygenated aCSF at 32 °C at a flow rate of 2 mL/min for 20 min to stabilize the slices. Neurons were visualized using an upright infrared differential interference contrast (IR-DIC) microscope (Nikon, Tokyo, Japan) equipped with a 40× water immersion objective. For EPSC recordings, 10 μM bicuculline was added to aCSF to record spontaneous excitatory postsynaptic currents (sEPSCs). For IPSC recordings, 10 μM CNQX and 50 μM D-APV were added to aCSF to record spontaneous inhibitory postsynaptic currents (sIPSCs). All electrophysiological measurements were performed immediately after THz wave exposure.

To assess intrinsic excitability, neurons were stimulated by injecting a series of depolarizing pulses in the presence of 10 μM CNQX, 50 μM D-APV, and 10 μM bicuculline, and neuronal action potentials evoked by current stimulation were recorded.

The procedure for measuring paired pulse ratios was previously described. EPSCs were evoked by stimulating the vCA3-CA1 pathway, and pyramidal neurons were clamped at −70 mV in the presence of 10 μM bicuculline. The ratio was calculated as (second EPSC/first EPSC) × 100%.

The excitation–inhibition (E–I) ratio measurement procedure has been previously described [13]. Briefly, a concentric bipolar stimulating electrode (KedouBC, Suzhou, China) delivering 0.1 Hz stimulation was placed near the pyramidal layer of CA1 to obtain evoked synaptic responses. The brain slices were first perfused with aCSF to record the total postsynaptic current (PSC), and then the inhibitory postsynaptic current (IPSC) in the presence of 10 μM CNQX and 50 μM D-APV was recorded. Subsequently, 10 μM bicuculline was added to confirm that the remaining currents were inhibitory. The E–I ratio was calculated as (PSC − IPSC)/IPSC.

The methodology for long-term potentiation (LTP) recording has been previously described [14,15]. Patch pipettes with resistances of 1–2 MΩ were filled with basal aCSF. The brain slices were transferred to the recording chamber, positioned, and secured with a cover mesh. Prior to recording, the slices were perfused with oxygenated aCSF at 32 °C at a flow rate of 2–4 mL/min for at least 30 min to ensure slice stability. For LTP recording in the IL, the recording electrode was inserted into layer 2/3, and the stimulating electrode was placed in layer 5. In the vCA1, the recording electrode was positioned in the stratum radiatum of vCA1, whereas the stimulating electrode was inserted into the Schaffer collaterals of vCA3, also within the stratum radiatum. The depth, stimulation intensity, and positioning of both electrodes were adjusted to optimize the amplitude of the field excitatory postsynaptic potential (fEPSP). Once the maximum fEPSP amplitude was achieved, the stimulation intensity was reduced to 50%, and a 20 min baseline recording was obtained. If the fEPSP amplitude remained below 0.1 mV after repeated adjustments, the brain slice was deemed nonviable and discarded. Electrical stimulation was conducted using a stimulator (AM SYSTEM, Sequim, USA) to induce LTP by applying three 1 s trains of high-frequency stimulation (HFS) at 100 Hz, with 20 s intervals between each train. Following HFS, fEPSP were recorded for an additional 30 min to monitor LTP.

All electrical signals were acquired using a Multiclamp 700B amplifier (Molecular Devices, San Jose, CA, USA) and digitized by a Digidata 1440A (Molecular Devices, San Jose, CA, USA) at a sampling rate of 20 kHz. Data acquisition and analysis were performed using Clampex 10 software (Molecular Devices, San Jose, CA, USA).

### 2.7. Fluorescence In Situ Hybridization

Fluorescence in situ hybridization was conducted via an RNAscope Multichannel Fluorescence Kit (ACD, Newark, NJ, USA) following the manufacturer’s instructions [16]. The samples were initially fixed with 4% paraformaldehyde (PFA) for 30 min at room temperature, subsequently washed with phosphate-buffered saline (PBS), and then dehydrated in a series of graded ethanol solutions. The samples were treated with hydrogen peroxide to eliminate endogenous peroxidase activity and incubated with proteinase III at 40 °C for 30 min to expose the target RNA. RNAscope probes specific to the RNA targets were then applied and incubated in the HybEZ hybridization system for 2 h under wet conditions. Following signal amplification, different channels were labeled with distinct fluorescent dyes. To prevent cross-contamination, an HRP blocking reagent was applied after staining, and the slides were mounted with antifade mounting medium containing DAPI. The fluorescent signals from different channels were captured using confocal microscopy, which revealed the spatial and quantitative distributions of the target RNAs.

### 2.8. Western Blot Analysis

Protein expression quantification was conducted using the Jess automated protein analysis system (Bio-Techne, Minneapolis, MN, USA), which provides a streamlined and accurate approach to protein level measurement. Proteins were extracted from mouse brain slices containing the vCA1 region using RIPA lysis buffer supplemented with protease inhibitors, and protein concentrations were measured using the BCA protein quantification method (Thermo, Waltham, MA, USA). Following sample preparation, proteins were loaded into the Jess system, which automatically performed electrophoretic separation, antibody incubation, washing, detection, and data analysis. The primary antibodies used were rabbit anti-VAMP2 (1:10,000), anti-SNAP25 (1:1000), anti-Syntaxin1 (1:1000), anti-SYN (1:5000), anti-GluA1 (1:1000), anti-GluA2 (1:1000), anti-GluN1 (1:1000), anti-GluN2A (1:1000), anti-GluN2B (1:1000), anti-EAAT1 (1:1000), anti-EAAT2 (1:1000), anti-GS (1:1000), anti-Glutaminase (1:1000), anti-CaMKII (1:1000), anti-PSD95 (1:1000), and anti-GAPDH (1:1000) (all from Abcam, Cambridge, UK) antibodies. The secondary antibodies selected for species compatibility were provided as ready-to-use solutions by Jess. Chemiluminescent signals were captured automatically by a high-sensitivity imaging device, and electropherograms were generated for each sample. Target protein expression levels were quantified by analyzing the peak areas of the electrophoretic signals. The data were processed and statistically analyzed using Compass for Simple Western 6.2.0, and the results were presented as graphical outputs for further interpretation.

### 2.9. Immunofluorescence Staining

For immunofluorescence of acute brain slices, brain slices were fixed in 4% PFA at 4 °C for 12 h. Following fixation, the slices were blocked in PBS containing 10% goat serum and 0.5% Triton X-100 at room temperature for 4 h. The slices were then incubated overnight at 4 °C with primary antibodies, including rabbit anti-c-Fos (Abcam, Cambridge, UK). The next day, the slices were incubated overnight at 4 °C with fluorescent secondary antibodies conjugated to Alexa Fluor 488 or Alexa Fluor 546 (1:2000, Invitrogen, Waltham, MA, USA) and DAPI. Afterward, the sections were washed three times with PBS, mounted on slides, and coverslipped with antifade mounting medium (Invitrogen, Waltham, MA, USA).

For immunofluorescence, the frozen sections were fixed in 4% PFA for 15 min at room temperature. Following fixation and blocking for 4 h at room temperature, the sections were incubated overnight at 4 °C with primary antibodies, including rabbit anti-c-Fos, rabbit anti-CaMKII and rabbit anti-VGAT antibodies (all from Abcam, Cambridge, UK). The following day, the sections were incubated with fluorescent secondary antibodies conjugated to Alexa Fluor 488 or Alexa Fluor 546 (1:2000, Invitrogen, Waltham, MA, USA) at room temperature for 2 h. After being washed three times in PBS, the sections were coverslipped with antifade mounting medium containing DAPI (Invitrogen, Waltham, MA, USA). Confocal microscopy (Oxford Instruments, Abingdon UK) was used for imaging. Cell counts and mean optical densities were analyzed using ImageJ software (ImageJ 1.x).

### 2.10. HD-MEA

The MaxOne HD-MEA system (Maxwell, Zurich, Switzerland) comprises 26,400 electrodes and 1024 channels integrated into a 4 × 2 mm MaxOne chip, facilitating the recording of synchronous firing activities in neurons. To achieve optimal contact between the electrodes and brain slices, the slices were carefully positioned on the HD-MEA and secured with mesh covers to minimize movement. The slices were continuously perfused with oxygenated aCSF at a pH of 7.4 and maintained at a temperature of 33 ± 1 °C. Synchronous neuronal firing was induced using the GABA_A_ receptor antagonist bicuculline. The activity scanning analysis module was employed to assess the spontaneous activity of neurons using a sparse 7× configuration, which involved seven different setups for 30 s intervals each. The system automatically identifies active neurons on the basis of their firing frequency and amplitude. The network analysis module was subsequently used to monitor the selected active neurons within the target area for a period of 5 min. MATLAB R2020a was subsequently used to process the raw data, analyze the temporal and spatial distributions of synchronous firing activities, quantify the proportion of peaks within bursts, and calculate the synchronous firing rate.

### 2.11. Molecular Dynamics Simulations

In order to study the effect of terahertz waves on the transmembrane domain (TMD) of AMPAR and its ion transport process, molecular dynamics simulation was used, and umbrella sampling was combined to calculate the mean free energy change (PMF) of Na^+^ through the selective filter (SF) [17]. The TMD structure was extracted from the RCSB Protein Data Bank, and the membrane protein-solvent system embedded in the DPPC biological membrane was constructed using CHARMM-GUI [18]. The equilibrium simulation was performed using the CHARMM36 force field at 1 bar and 310 K [19]. Multiple windows were set in the SF region for umbrella sampling, the Na^+^ position was controlled, and a 50 ns MD simulation was run. The PMF curve was calculated by weighted histogram analysis to evaluate the effect of terahertz waves on Na^+^ permeability. In addition, the B-factor changes of key residues (Gln586, Gln587, Gly588) were calculated to evaluate the effect of terahertz waves on the flexibility of the SF region, thereby exploring its regulatory effect on ion permeability.

### 2.12. Slice Calcium Imaging

Brain slices expressing genetically encoded calcium indicators were placed in a perfusion chamber and continuously perfused with oxygenated aCSF at a constant flow rate of 2 mL/min. Calcium signals were recorded using a charge-coupled device (CCD) camera (Mightex, Toronto, Canada), with the fluorescent calcium indicator excited at a wavelength of 470 nm. Neuronal calcium activity was electrically stimulated using parameters of 100 Hz frequency, 0.1 mA amplitude, 0.45 ms pulse duration, and 2 s duration, as previously described [20]. Time series images capturing calcium dynamics were obtained with a high-speed CCD camera. The fluorescence data were analyzed using IAA (Mightex, Toronto, Canada), and the changes in the calcium signals were quantified as ΔF/F, where ΔF represents the change in fluorescence intensity and F denotes the baseline fluorescence intensity at each time point.

### 2.13. In Vitro Optogenetics

Mice injected with rAAV-CaMKIIa-ArchT-EGFP in the vCA1 for 2 weeks were anesthetized, and IL brain slices were prepared. Under 470 nm excitation, nerve endings projecting from the vCA1 to the IL were identified. The stimulation area was selected using an in vitro optogenetic system (Mightex, Toronto, Canada), and the photosensitive ArchT proteins on the nerve endings were stimulated with 560 nm light to inhibit their function. Based on published study, the laser power was set to 10 mW, and continuous wave stimulation was applied [21]. Patch-clamp recordings were performed following the standard protocol previously described. First, the EPSCs of neurons in the light-off state were recorded for 120 s, and then the EPSCs of neurons in the light-on state were recorded for 120 s. The EPSC frequency reflects the strength of vCA1-IL neural projections. On the basis of the exposure to structured THz waves and 560 nm light stimulation, the brain slices were divided into four experimental groups: C, C + Light, T, and T + Light.

### 2.14. In Vivo Optogenetics

Prior to the experiment, the wired optogenetics system was activated, and the optical fiber was connected. The light source was turned on and preheated for 20 min. Initially, the waveform was set to direct current mode, and the power was adjusted to 10 mW. The waveform was subsequently switched to square wave mode, and the light activation parameters were configured as follows: 465 nm blue light stimulation with a frequency of 20 Hz, pulse width of 5 ms, and laser activation for 2 s every 2 min. The optical fiber was then coupled to the ceramic ferrule, and light stimulation was administered to the mice during the behavioral experiments.

### 2.15. Behavioral Experiments

For the open field test, the mice were placed in an open field arena and monitored for 5 min. Their exploratory behavior, including the time spent in the central area and their movement speed, was recorded. After each session, the arena was thoroughly cleaned to eliminate odors, ensuring a consistent experimental environment for subsequent mice.

In the novel object recognition test, during the learning phase, each mouse was placed in the open field arena facing away from two objects and allowed to explore freely for 5 min. 30 min after the learning phase, the test phase was initiated by replacing one object with a new one of a different shape while maintaining its original position. The mouse was then allowed to explore freely for an additional 5 min. The discrimination index was calculated as (time spent exploring the new object/total exploration time) × 100%.

For the Y-maze novel arm test, during the training phase, the mice were placed in the starting arm of the Y-maze and allowed to explore the two open arms for 8 min, and the third arm (novel arm) was closed. In the test phase, the novel arm was opened, and the mice could freely explore all three arms for 5 min. The discrimination index was calculated as (number of entries into the novel arm/total number of arm choices) × 100%.

In the Y-maze spontaneous alternation test, the mice were placed at the midpoint of the Y-maze and allowed to explore freely for 5 min. After 60 min of THz waves exposure for seven consecutive days, the Y-maze test was performed again. A correct alternation was defined as entering three different arms consecutively. The spontaneous alternation rate was calculated as [number of correct spontaneous alternations/(total number of arm entries − 2)] × 100%.

Finally, in the T-maze delayed unpaired task, the mice were subjected to food restriction until their body weight decreased by 15%. Ten trials were conducted daily, each consisting of a sampling period, a delay period, and a selection period. During the sampling period, the mice were forced to enter an arm to receive a reward and then returned to the starting position. The selection period, separated by a 10 s delay, allowed the mice to freely choose an arm. If the mouse entered the nonselection arm, it received a reward; if it entered the selection arm, it received no reward. The T-maze accuracy rate was calculated as (number of correct choices/total number of trials) × 100%.

### 2.16. ATAC-Seq Analysis

Quality control (QC) was performed before and after read trimming using FastQC (v.0.11.9), and no issues were highlighted from the QC process. Subsequent read alignment and post alignment filtering were performed in accordance with the ENCODE project’s “ATAC-seq Data Standards and Prototype Processing Pipeline” for the data. In brief, the reads were mapped to the mm10 assembly of the mouse genome via Bowtie2 (v.2.3.5.1). The resulting sequence alignment map (SAM) files were compressed to the binary alignment map (BAM) version, in which SAMtools (v.1.11) was used to filter reads that were unmapped, mate unmapped, not primary alignment or failed platform quality checks. From the processed BAM files, coverage tracks in bigWig format were generated using deepTools bamCoverage (v.3.5.1), and peaks were called using MACS2 (v.2.2.7.1).

Analysis of ATAC-seq data for differential accessibility was carried out in R (v.4.1.3) using the DiffBind package (v.3.4.11) with default parameter settings. Differential accessibility across samples was calculated using the negative binomial distribution model implemented in DEseq2 (v.1.34.0).

### 2.17. Hi-C Analysis

The paired-end reads were processed using HiC-Pro (v.3.1.0) (https://github.com/nservant/HiC-Pro). The A/B compartments were calculated via homer v5.1 from normalized Hi-C allValidPairs data. Then, we obtained hierarchical TADs from normalized Hi-C matrix data via OnTAD v1.2. In brief, OnTAD first uses an adaptive local minimum search algorithm to identify candidate TAD boundaries. Then, OnTAD assembles TADs from the candidate boundaries using a recursive algorithm. We take the 10 kb resolution Hi-C interaction matrix as input to calculate the hierarchical TAD structure with the parameters—penalty 0.1—maxsz 200. Stable loops were identified using HiCCUPS from Juicer (v.1.22.01) at 5 kb resolution for the highest-quality contacts, and 836/713 loops were called from C/T samples. Hichcupsdiff calculated that 28 loops were gained and that 46 loops were lost after terahertz radiation.

We used the “TH score” as defined by Du et al. in 2021 to assess the TAD nesting level of genes [22]. We calculated the “TH score” for each gene using Bedtools 2.31.1 with the “coverage” command on two given BED files describing gene regions and TAD regions. We then calculated the differential TH score (Diff-TH score = THT − THC) of each gene between normal and terahertz tissues. Genes with Diff-TH scores >2.5 were considered significantly upregulated, and genes with Diff-TH scores <2.5 were considered significantly downregulated.

### 2.18. Sn RNA-Seq Analysis

QC was performed using the Cell Ranger package v.7.2.0. The FASTQ formats were aligned with the mm10 reference genome. The aligned reads were filtered by cell barcodes and the unique molecular identifier (UMI) to establish filtered gene–barcode matrices. The preprocessed digital gene expression (DGE) profiling matrices were imported utilizing the Seurat package (v.5.0.1) in R (v.4.1.3). The data were screened to detect cells with 500 to 7500 genes (500 < nFeature_RNA < 7500), a sum of expression amount per cell less than 50,000 (nCount_RNA < 50,000), and mitochondrial genes less than 5% (percent.mt < 5%). The snRNA-seq datasets were subsequently analyzed using Seurat as described previously. In brief, cells were grouped by cell clusters with UMAP as determined by the function FindClusters of Seurat, which calculated 10 principal component analysis (PCA) components with a resolution of 1.0, leading to 25 clusters.

### 2.19. Statistical Analysis

Data analysis was performed using SPSS 25 software, and statistical graphs were drawn using GraphPad Prism 8.0 software. Unpaired two-tailed *t*-tests were used to compare two groups in the case of a normal sample distribution. Repeated measures analysis of variance was used to compare differences between the groups for the Y-maze spontaneous alternation test and the T-maze delayed unpaired task, and *p* < 0.05 was considered statistically significant.

## 3. Results

### 3.1. Glutamatergic Neurons Were Activated After THz Exposure

**Figure 1:**
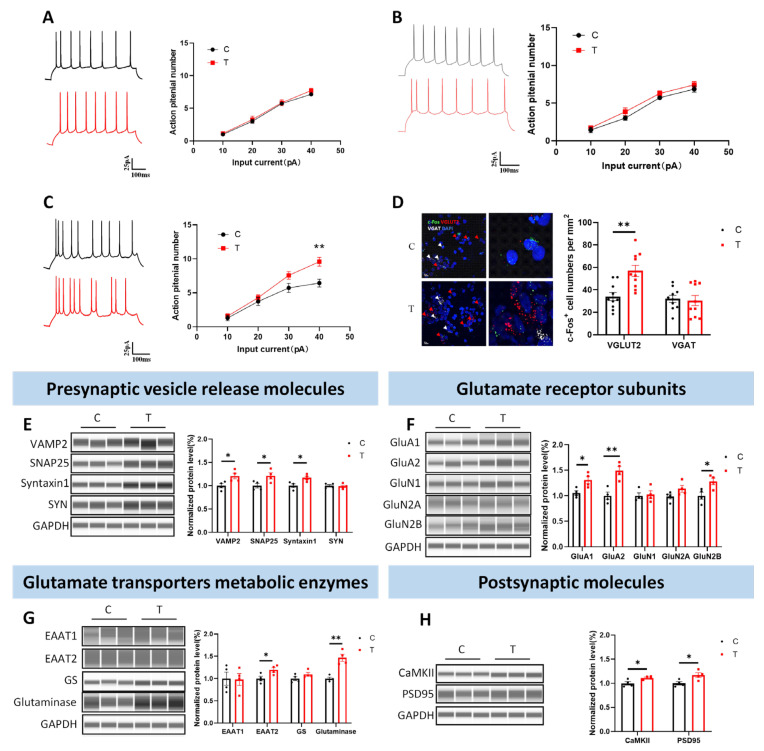
THz waves increase glutamatergic neuron activity. (**A**–**C**) Action potentials of primary hippocampal neurons exposed to THz waves for 6, 30, and 60 min; (**D**) Fluorescence in situ hybridization of c-Fos, VGLUT2, and VGAT after THz waves exposure; (**E**–**H**) Western blot analyses of vesicle release-related molecules, glutamate receptor subunits, glutamate transporter metabolic enzymes, and postsynaptic molecules in primary hippocampal neurons. * Represents *p* < 0.05, ** Represents *p* < 0.01.

First, we detected the temperature change during 0.152 THz exposure and found that the temperature increase after 60 min of exposure was less than 1 °C (Figure S2A). To determine the THz waves exposure conditions that affect the intrinsic excitability of primary hippocampal neurons, we measured action potentials following 6, 30, and 60 min of exposure. No significant changes in neuronal action potentials were observed after 6 min (Figure 1A) or 30 min (Figure 1B) of THz exposure. However, after 60 min of exposure, the number of action potentials generated by neurons in the T group at 40 pA was significantly greater than that generated by neurons in the C group (Figure 1C). These findings suggested that 0.152 THz exposure for 60 min could increase the intrinsic excitability of primary hippocampal neurons, prompting further studies under these conditions. Based on this result, the subsequent experiments selected the exposure condition of 0.152 THz irradiation for 60 min to further study the effects of THz waves on neuronal function and its potential mechanisms.

To explore the effects of THz waves on neuronal activity at the molecular level, we labeled glutamatergic neurons (VGLUT2), GABAergic neurons (VGAT), and an immediate early gene marker (c-Fos) using RNAscope fluorescence in situ hybridization. Following THz waves exposure, the number of c-Fos^+^/CaMKII^+^ neurons significantly increased, whereas no significant change was observed in the proportion of c-Fos^+^/VGAT^+^ neurons (Figure 1D). These findings indicated that THz waves predominantly increased the activity of glutamatergic neurons rather than GABAergic neurons.

To investigate the molecular mechanisms underlying these changes, we assessed the expression of molecules involved in glutamate metabolism by Western blot. Compared with those in the C group, the expression levels of VAMP2, SNAP25, and Syntaxin1 were significantly greater in the T group, whereas the synaptophysin level remained unchanged (Figure 1E). Compared with those in the C group, the expression levels of GluA1, GluA2, and GluN2B were significantly greater in the T group, whereas the levels of GluN1 and GluN2A did not change significantly (Figure 1F). Compared with those in the C group, the expression levels of EAAT2 and glutaminase were elevated in the T group, whereas the expression levels of EAAT1 and GS were not significantly different (Figure 1G). Finally, the expression levels of PSD95 and CaMKII in the T group were significantly greater than those in the C group (Figure 1H).

### 3.2. THz Waves Increased the Activity and Promoted the Synaptic Plasticity of IL and vCA1 Neurons

**Figure 2:**
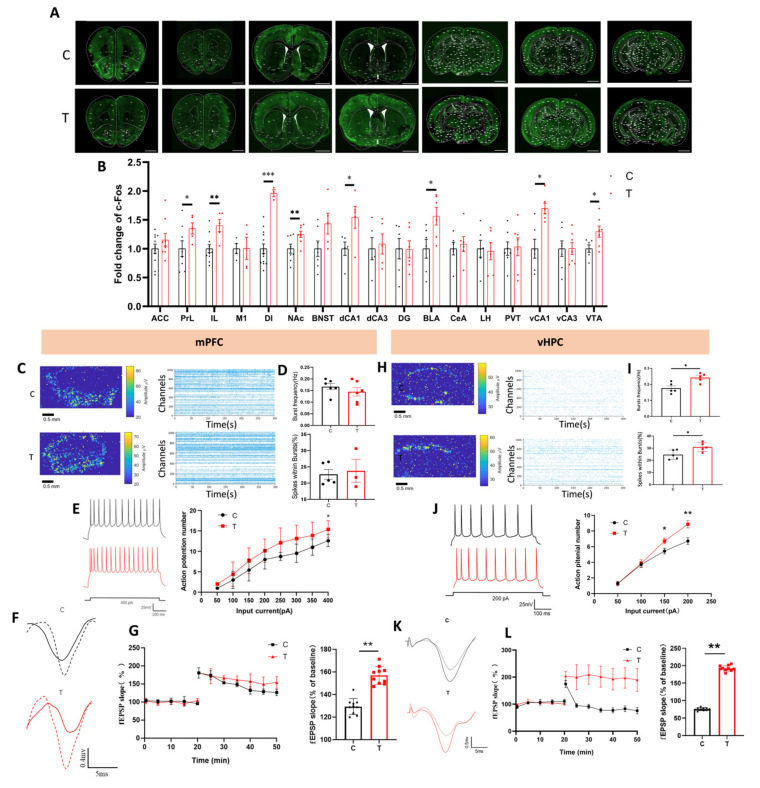
THz waves increase IL and vCA1 neuron activity and promote synaptic plasticity. (**A**) c-Fos immunofluorescence staining, scale bar = 1000 μm; (**B**) c-Fos^+^ cell number statistical analysis; (**C**) IL neuron firing raster plots and heatmaps; (**D**) IL neuron synchronous firing frequency and spike ratio statistical analysis; (**E**) IL neuron action potential; (**F**) IL neuron fEPSP representative trace; (**G**) IL neuron fEPSP statistical analysis; (**H**) vCA1 neuron firing raster plots and heatmaps; (**I**) vCA1 neuron synchronous firing frequency and spike ratio statistical analysis; (**J**) vCA1 neuron action potential; (**K**) vCA1 neuron fEPSP representative trace; (**L**) vCA1 neuron fEPSP statistical analysis. * represents *p* < 0.05, ** represents *p* < 0.01, *** represents *p* < 0.001.

The effects of THz waves on neuronal activity may vary across different brain regions. To investigate this, we assessed neuronal activity in 16 brain regions by c-Fos immunofluorescence staining after THz waves exposure (Figure 2A). Compared with that in the C group, the number of c-Fos^+^ cells increased significantly in 7 brain regions, including the IL and vCA1, both of which were critical regions for working memory (Figure 2B).

To further examine the effects of THz waves on neuronal electrical activity, we used a HD-MEA to record the network firing activity of IL neurons. The raster plots and heatmaps derived from these recordings are shown in Figure 2C. No significant changes in the synchronous firing activity of IL neurons were observed after THz exposure, as the synchronous firing frequency and spike ratio remained unchanged (Figure 2D).

We also assessed the intrinsic excitability of IL neurons by injecting a series of depolarizing current pulses and measuring the resulting action potentials. Compared with that in the C group, the number of action potentials at 400 pA was significantly greater in the T group (Figure 2E).

To evaluate the effects of THz waves on synaptic plasticity, we conducted LTP recording in the IL (Figure 2F). The fEPSP trace is shown in Figure 2F. We observed that the fEPSP slope in the T group decreased more slowly after HFS than did that in the C group. Quantitative analysis of the fEPSP slope from 40 to 50 min revealed that the fEPSP slope significantly increased in the T group (Figure 2G).

Similar experiments were conducted to assess the network firing activity, action potentials, and LTP of vCA1 neurons. After THz exposure, the network firing activity of vCA1 neurons was significantly increased, with increases in the burst firing frequency and spike ratio (Figure 2H,I). The number of action potentials at both 150 pA and 200 pA was significantly greater in the T group (Figure 2J). Additionally, THz exposure promoted synaptic plasticity in the vCA1, as manifested by an increase in the fEPSP slope (Figure 2K,L).

In summary, THz waves increased neuronal activity in the IL and vCA1, regions critical for working memory, by increasing network firing activity and intrinsic excitability and promoting synaptic functional plasticity.

### 3.3. THz Waves Increased Glutamatergic Synaptic Transmission in IL and vCA1 Neurons

**Figure 3:**
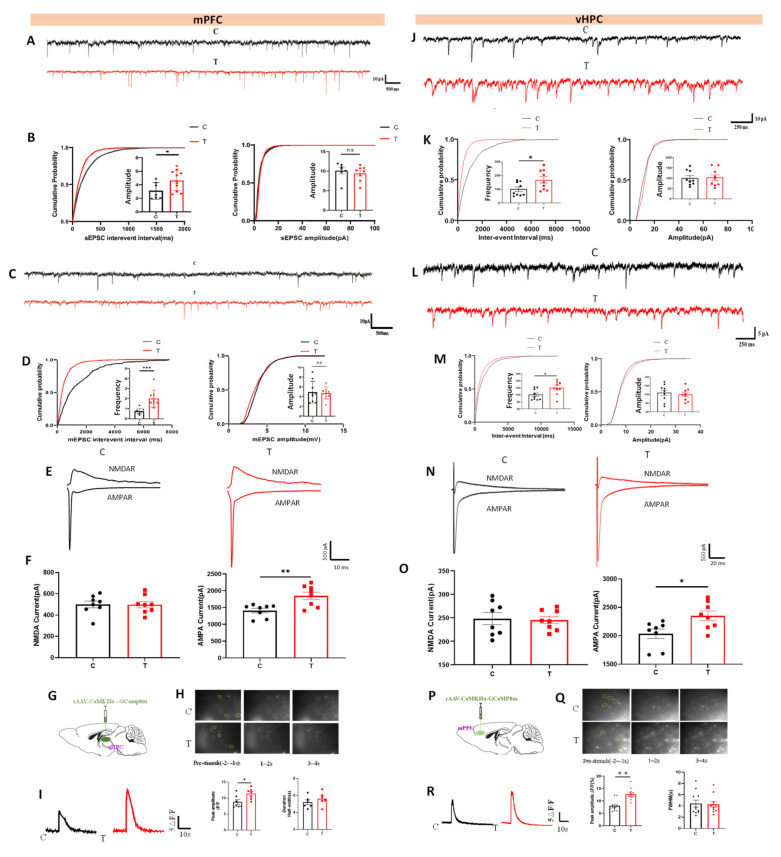
THz waves enhance IL and vCA1 neuron glutamatergic synaptic transmission. (**A**) sEPSC traces of IL neurons; (**B**) Quantitative analysis of frequency and amplitude of sEPSC of IL neurons; (**C**) sIPSC traces of IL neurons; (**D**) Quantitative analysis of frequency and amplitude of sIPSC of IL neurons; (**E**) Glutamate receptor currents in IL neurons; (**F**) Quantitative analysis of glutamate receptor currents; (**G**) Schematic diagram of viral injection for calcium imaging in the IL region; (**H**) IL neurons labeled with genetically encoded calcium indicators; (**I**) Statistical analysis of calcium activity in IL neurons; (**J**) sEPSC traces of vCA1 neurons; (**K**) Quantitative analysis of frequency and amplitude of sEPSC of vCA1 neurons; (**L**) sIPSC traces of vCA1 neurons; (**M**) Quantitative analysis of frequency and amplitude of sIPSC of vCA1 neurons; (**N**) Glutamate receptor currents in vCA1 neurons; (**O**) Quantitative analysis of glutamate receptor currents; (**P**) Schematic diagram of viral injection for calcium imaging in the vCA1 region; (**Q**) vCA1 neurons labeled with genetically encoded calcium indicators; (**R**) Statistical analysis of calcium activity in vCA1 neurons. * represents *p* < 0.05, ** represents *p* < 0.01, *** represents *p* < 0.001.

To investigate the effects of THz waves on synaptic transmission in IL neurons, we recorded sEPSCs and sIPSCs. In the IL, the frequency of sEPSCs in the T group was significantly greater than that in the C group, whereas the amplitude remained unchanged (Figure 3A,B). There were no significant differences observed in the frequency or amplitude of sIPSCs (Figure 3C,D). Additionally, we examined glutamate receptor currents in IL neurons. The AMPAR current in the T group was significantly elevated, whereas the NMDAR current did not significantly change (Figure 3E,F). In addition, molecular dynamics simulations found that the THz electric field changed the free energy curve of Na^+^ passing through the SF of the AMPA receptor TMD, reducing the minimum PMF and increasing the potential energy difference, making the Na^+^ permeation process more spontaneous and reducing energy requirements (Figure S3A). Further analysis found that the B-factor of key residues Gln586, Gln587 and Gly588 increased significantly, indicating that THz waves enhance the flexibility of the SF region, help optimize the ion channel structure, and thus improve the Na^+^ transport efficiency (Figure S3B).

Neuronal calcium signaling, which is crucial for processes such as synaptic plasticity and neurotransmitter release, was also assessed [23]. Since both the IL and vCA1 predominantly contain glutamatergic neurons, we used genetically encoded calcium indicators to monitor calcium activity in these regions. A schematic of virus injection into the IL is shown in Figure 3G, with virus-labeled glutamatergic neurons visible under a CCD camera. The recorded calcium signal traces are presented in Figure 3H. Compared with those in the C group, the calcium event amplitude in the glutamatergic neurons in the T group was significantly greater, whereas the calcium event half-width remained unchanged (Figure 3I).

A similar approach was used to record postsynaptic currents, glutamate receptor currents, and calcium activity in vCA1 neurons. In the T group, the frequency of sEPSCs in vCA1 neurons was increased, with no significant change in amplitude (Figure 3J,K). Similarly, the frequency of sIPSCs in vCA1 neurons increased, but the amplitude did not change significantly (Figure 3L,M). Consistent with the IL results, AMPAR currents in vCA1 neurons were significantly elevated in the T group, whereas NMDAR currents remained unchanged (Figure 3N,O). Additionally, THz waves exposure increased calcium activity in vCA1 glutamatergic neurons, which manifested as an increase in the amplitude of calcium events, with no significant change in the half-width of calcium events (Figure 3P–R).

After exposure to THz waves, both excitatory and inhibitory synaptic transmission in vCA1 neurons were increased. To assess the impact of THz waves on the excitation-inhibition balance, we measured the EPSC-IPSC ratio, which revealed that THz waves significantly increased this ratio (Figure S4A,B). These results suggested that the primary effect of THz waves was the increase in excitatory synaptic transmission. We also evaluated the paired-pulse ratio (PPR), an indicator of presynaptic vesicle release, and detected a decrease in the PPR (Figure S4C,D), indicating that THz waves could increase presynaptic vesicle release.

Finally, Western blot analysis of glutamate metabolism-related molecules in the IL revealed significant increases in the expressions of vesicle release-related molecules (VAMP2, SNAP25, and Syntaxin1) (Figure S5A), glutamate receptor subunits (GluA1, GluA2 and GluN2B) (Figure S5B), glutamate transporter enzymes (EAAT1, EAAT2 and Glutaminase) (Figure S5C), and postsynaptic signaling molecules (CaMKII and PSD95) (Figure S5D) in the T group compared with those in the C group. Similarly, in vCA1 neurons, the expression of vesicle release-related molecules (VAMP2, SNAP25, and Syntaxin1) (Figure S5E), glutamate receptor subunits (GluA1, GluA2) (Figure S5F), glutamate transporters and metabolic enzymes (EAAT2, GS, and Glutaminase) (Figure S5G), and postsynaptic signaling molecules (CaMKII and PSD95) (Figure S5H) were also significantly greater in the T group than in the C group.

To investigate the effects of THz waves on the function of vCA1-IL glutamatergic projections, rAAV-CaMKIIa-ArchT-EGFP was injected into the vCA1 region. This allowed for selective light-mediated inhibition of vCA1-IL glutamatergic projections, and the sEPSCs of IL neurons were subsequently recorded to assess the impact of THz waves exposure on these projections (Figure S6A). The virus injection sites were confirmed, and fluorescent marker proteins were successfully expressed in both the vCA1 and IL regions (Figure S6B). After THz waves exposure, the frequency of sEPSCs increased. However, when vCA1-IL glutamatergic projections were inhibited by light, there were no significant differences in sEPSC frequency compared with those in Group C (Figure S6C,D). These findings suggested that THz waves can improve the function of vCA1-IL glutamatergic projections.

### 3.4. THz Waves Improved Spatial Working Memory in Mice

**Figure 4:**
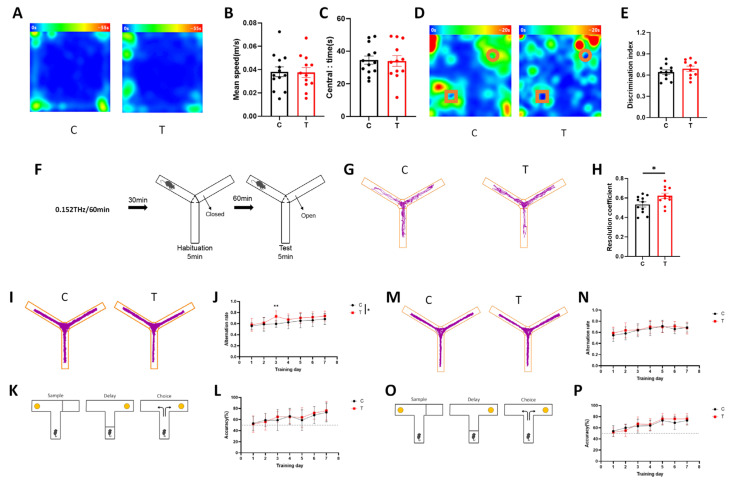
Effects of THz waves on the working memory of mice. (**A**) Heatmap of the open field traces of the mice after THz waves exposure to the IL; (**B**) Statistical analysis of average speed; (**C**) Statistical analysis of central area time; (**D**) Heatmap of the traces of the mice in the novel object recognition experiment after THz waves exposure to the IL; (**E**) Statistical analysis of the discrimination index; (**F**) Schematic diagram of the novel arm experiment in the Y-maze after THz waves exposure to the IL; (**G**) Graph of the mouse movement trace in the novel arm experiment in the Y-maze; (**H**) Statistical analysis of the novel arm discrimination index; (**I**) Graph of the mouse trace in the spontaneous alternation experiment in the Y-maze after THz waves exposure to the IL; (**J**) Statistical analysis of the spontaneous alternation rate; (**K**) Schematic diagram of the delayed unpaired task in the T-maze after THz waves exposure to the IL; (**L**) Statistical analysis of task accuracy; (**M**) Graph of the mouse trace in the spontaneous alternation experiment in the Y-maze after THz waves exposure to the vCA1; (**N**) Statistical analysis of the spontaneous alternation rate; (**O**) Schematic diagram of the delayed unpaired task in the T-maze after THz waves exposure to the vCA1; (**P**) Statistical analysis of task accuracy. * represents *p* < 0.05, ** represents *p* < 0.01.

After the transparent skull protocol was performed, the mice were subjected to behavioral experiments beginning 7 d after surgery. Initially, the transmittance of THz waves through the transparent skull was measured at 86.1% using a THz wave power meter. After 60 min of THz waves exposure, the temperature increase on the skull surface was less than 1 °C (Figure S1B). Immunofluorescence staining for c-Fos and neuronal markers (CaMKII and VGAT) in the IL revealed an increase in the number of c-Fos^+^/CaMKII^+^ neurons after THz waves exposure (Figure S7A,B), whereas the number of c-Fos^+^/VGAT^+^ neurons remained unchanged (Figure S7C,D), indicating that THz waves specifically increase the activity of IL glutamatergic neurons. To minimize anxiety induced by the experimenter, the mice were gently handled and acclimated for 3 d. After THz waves exposure, the mice were sequentially subjected to the open field test, novel object recognition test, and Y-maze test. The open field test revealed no significant differences in locomotion speed or time spent in the central area (Figure 4A–C), suggesting that THz waves did not affect locomotor activity or anxiety levels. In the novel object recognition test, THz waves exposure did not significantly alter the discrimination index (Figure 4D,E). However, in the Y-maze test, the novel arm discrimination indices of the T group were greater than those of the C group (Figure 4F–H), indicating improved spatial working memory.

To investigate the effects of long-term THz waves exposure on working memory acquisition speed, the mice were exposed to THz waves for consecutive 7 d, 1 h per day. After each exposure, Y-maze spontaneous alternation and T-maze delayed nonmatched tasks were performed. The results revealed an increase in the spontaneous alternation rate in the Y maze within the IL after 7 d of THz waves exposure, with the T group outperforming the C group by 3 d (Figure 4I,J). No significant changes were observed in the T-maze performance over the 7 d period (Figure 4K,L) or in the vCA1 performance in either maze test (Figure 4M–P). These findings suggested that long-term THz waves exposure to the IL improved spatial working memory, as manifested by increased Y-maze spontaneous alternation rates.

Additionally, we compared the neuromodulatory effects of THz waves using optogenetics. To evaluate the effects of optogenetic activation of vCA1-IL glutamatergic projections and THz waves exposure of the IL region on working memory, optogenetic activation virus (rAAV-CaMKIIa-ChrimsonR-mCherry) or control virus (rAAV-CaMKIIa-mCherry) was injected into the vCA1, and ceramic fibers were implanted in the downstream IL for optogenetic stimulation (Figure S8A). Verification of viral expression revealed red fluorescent protein in both the vCA1 and IL regions (Figure S8B). Compared with that in the C + mCherry group, the spontaneous alternation rate in the Y-maze increased in both the C + ChrimsonR and T + mCherry groups, with the C + ChrimsonR group showing a higher alternation rate than the C + mCherry group on the 2 d (Figure S8C,D). Similarly, to compare the neuromodulatory effects of optogenetically activated IL glutamatergic neurons and THz waves on working memory, we injected either the optogenetic activation virus or control virus into the IL and implanted ceramic fibers for optogenetic stimulation (Figure S8E). Red fluorescent protein expression was observed in the IL (Figure S8F). Compared with that in the C + mCherry group, the Y-maze spontaneous alternation rate in the Y-maze was greater in both the C + ChrimsonR and T + mCherry groups. On d 4, the alternation rate in the C + ChrimsonR group was significantly greater than that in the C + mCherry group (Figure S8G,H). These results suggested that THz waves could achieve similar neuromodulatory effects comparable to those of optogenetics.

### 3.5. THz Waves Induced Changes in the Neuronal Epigenome and Genome

**Figure 5:**
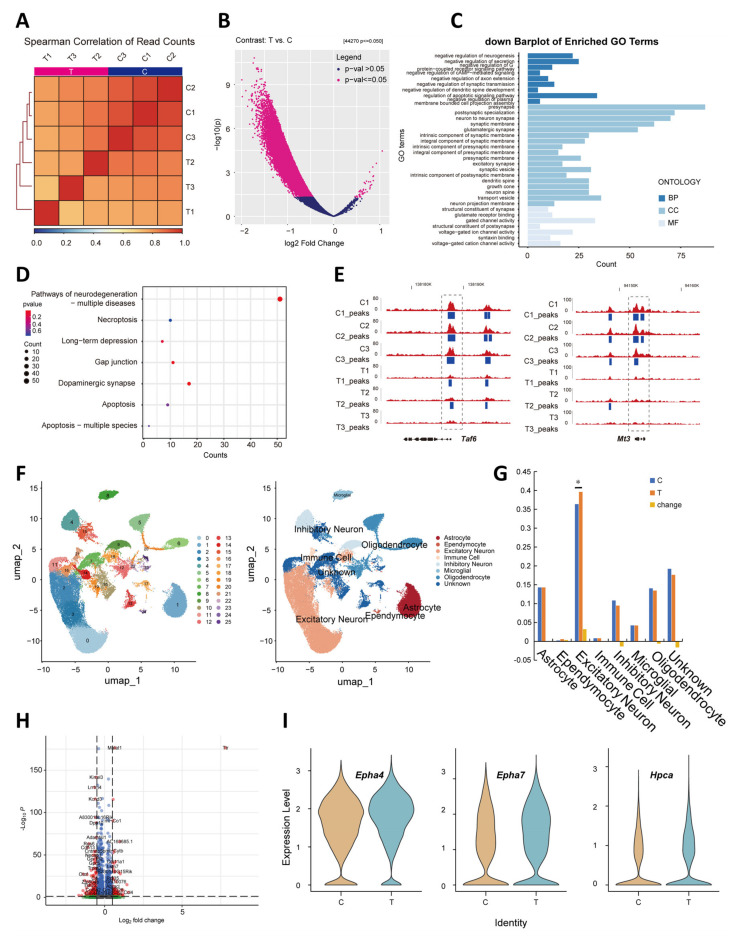
Effects of THz waves on neuronal chromatin accessibility and the genome. (**A**) Spearman correlation heatmap of read counts in the C and T groups. (**B**) Volcano plot of T-C contrast in ATAC-seq. (**C**) Bar plot of enriched GO terms from ATAC down peaks in the T group. (**D**) Bubble plot of enriched KEGG terms from ATAC down peaks in the T group. (**E**) ATAC-seq and peak density levels are shown at representative promoters. (**F**) snRNA-seq of brain tissue samples obtained from mice. Uniform manifold approximation and projection (UMAP) clustering of single-cell transcriptomes colored according to PC clusters (**left**) and significant cell-type clusters (**right**). (**G**) Percent bar plot of cell counts according to cell type clusters. (**H**) Volcano plot of the T-C contrast in the excitatory neuron cluster. (**I**) Expression levels of upregulated genes are shown for a representative gene (Epha4, Epha7, Hpca) in the excitatory neuron cluster.

We used ATAC-seq to identify genomic regulatory elements after THz waves exposure in mouse brain tissue. This technique maps chromatin accessibility in brain tissue. Distinct chromatin accessibility profiles were observed after THz waves exposure. Using Bedtools, we calculated and visualized the Spearman correlations of the read counts, revealing that chromatin accessibility was altered by THz exposure (Figure 5A). The volcano plot illustrates the differentially accessible chromatin regions in response to THz waves exposure (Figure 5B). The x-axis represents the log2 fold change in chromatin accessibility, while the y-axis denotes the −log10(*p*-value), indicating statistical significance. Each point corresponds to a genomic region, with significantly differentially accessible peaks highlighted in pink. The asymmetric distribution of significant peaks toward the left side of the plot suggests a predominant downregulation of chromatin accessibility in the T group. We subsequently annotated the locations of these differential peaks and identified genes with significant chromatin accessibility changes in their promoter regions for further investigation. Gene Ontology (GO) and Kyoto Encyclopedia of Genes and Genomes (KEGG) enrichment analyses of these genes revealed that those with decreased chromatin accessibility were involved primarily in the negative regulation of neurogenesis and secretion pathways (Figure 5C,D). Genome browser views of the ATAC-seq profiles for Taf6 and Mt3 (Figure 5E) revealed a significant reduction in chromatin accessibility within the promoter regions in the T group.

PCA demonstrated high reproducibility among biological triplicates of Hi-C (Figure S9A), although dynamic changes in chromatin accessibility were detected between groups. To quantify the similarity between Hi-C interaction matrices at 10 kb resolution, we utilized HiCRep, the stratum-adjusted correlation coefficient (SCC) developed by Tao Yang [22] (Figure S9B). The analysis revealed high similarity between samples in the C and T groups. Homer was employed to calculate the principal component 1 (PC1) values, where regions with PC1 > 0 were classified as compartment A and those with PC1 < 0 were classified as compartment B. We assessed the magnitude of changes between different compartments before and after THz waves exposure and found that the proportion of compartment A was 54% before and 45% after THz waves exposure, whereas that of compartment B constituted 46% and 55%, respectively (Figure S9C). Only 1% of the compartments exhibited changes after THz waves exposure, transitioning between compartments A and B or vice versa. We calculated the differential TH score for each gene between the C and T groups. Genes with a Diff-TH score >2.5 were considered significantly upregulated, whereas those with a Diff-TH score <−2.5 were deemed significantly downregulated. GO enrichment analysis of these genes revealed that the upregulated genes were enriched primarily in cranial nerve morphogenesis and nerve development pathways (Figure S9D). Additionally, we utilized hiccupsdiff to identify gained and lost loops after THz waves exposure. Notably, a newly gained loop was observed near the Tshz3 gene after THz waves exposure (Figure S9E). Tshz3 is involved in the modulation of glutamatergic synaptic transmission and long-term synaptic potentiation. Related studies have shown that reduced expression of this gene and consequent caspase upregulation may be correlated with the progression of Alzheimer’s disease (AD) in human patients.

To gain insight into cell lineage changes after THz waves exposure, we performed snRNA-seq on mouse brain tissue to identify differences and similarities in cellular composition. The cells were filtered on the basis of having between 500 and 7500 genes detected (500 < nFeature_RNA < 7500), a total expression count per cell below 50,000 (nCount_RNA < 50,000), and less than 5% mitochondrial gene expression (percent.mt < 5%) (Figure S10A). We observed that nCount_RNA was negatively correlated with percent.mt and positively correlated with nFeature_RNA. The first 10 principal components (PCs) were selected for cell classification (Figure S10B–D). Unbiased clustering identified 25 subtypes, which were manually annotated into seven major cell groups: astrocytes, ependyocytes, excitatory neurons, immune cells, inhibitory neurons, microglia, and oligodendrocytes (Figure 5F). All cell subtypes were present in the mouse brain both before and after THz waves exposure, but their proportions varied (Figure 5G), with the proportion of excitatory neurons significantly increasing after THz waves exposure. Next, we investigated differentially expressed genes in excitatory neuron cell types after THz exposure (Figure 5H). Among these genes, Epha4, Epha7, and Hpca were upregulated after THz waves exposure, and these upregulated genes were involved mainly in synaptic plasticity, neural development, and signal transduction (Figure 5I).

## 4. Discussion

THz waves cover the vibration and rotation energy levels of many biomacromolecules, indicating that they might interact with and influence the structure and function of the nervous system. Increasing evidence has suggested that THz waves represent a novel neuromodulation method with promising therapeutic applications. A study used broadband THz waves with a power density of 2.4 mW/cm^2^ to treat patients with acute ischemic stroke for 6–10 consecutive sessions (22.5 min each), resulting in significant alleviation of neurological deficit symptoms [24]. Another study revealed that 34.5 THz irradiation of the mouse hippocampus CA3 region increased the binding between glutamate and GluN2B, thereby improving cognitive function in animals with post-traumatic stress disorder [25]. THz waves have also been explored for treating AD. Continuous exposure of AD model mice to 0.14 THz waves at an average power density of 25 mW/cm^2^ for 12 weeks improved cognitive function and alleviated pathological symptoms, including tau hyperphosphorylation, neuronal loss, neuroinflammation, and mitochondrial damage [26]. Previous study has shown that 0.14 THz can cultivate enhanced excitatory synaptic transmission and firing activity of neurons [27]. We performed molecular dynamics simulations near this frequency and found that at 0.152 THz, the ion transport function of AMPAR was significantly enhanced. Therefore, we chose this frequency to further study its effects on neuronal function and spatial working memory.

In the present study, we systematically investigated the effects of THz waves exposure on neuronal function and behavior, with a particular focus on glutamatergic neurons in the IL and vCA1. We conducted a series of experiments at the cellular, brain slice, and whole-animal levels to elucidate the mechanisms underlying THz wave-induced neuromodulation.

THz waves can increase neuronal activity. Studies have shown that 53.6 THz waves at 9 ± 0.5 mW could increase the proportion of c-Fos^+^ cells in mouse cortical neurons and induce neuronal spike firing [28]. While recent research has demonstrated that THz waves can increase neuronal activity in specific brain regions, the exact responses of different neuronal subtypes to THz waves remain unclear. Further research into the effects of THz waves on distinct neuronal subtypes will facilitate more precise neuromodulation. A key finding of this study was the identification of the exposure conditions for neuromodulatory effects of 0.152 THz waves, which, when applied for 60 min, increase the excitability of primary hippocampal neurons. In addition, in the high-frequency band, THz waves have been shown to improve learning and memory abilities, but the temperature increases above 1 °C [7]. Furthermore, we found that THz waves specifically increased the activity of glutamatergic neurons rather than GABAergic neurons. This effect was further evidenced by an increase in the levels of glutamate metabolic molecules in primary hippocampal neurons.

The IL and vCA1 are crucial brain regions involved in cognitive functions such as working memory, decision-making, and emotional regulation [29,30]. Moreover, synaptic plasticity and synaptic transmission form the basis of nervous system function, as well as learning and memory [31,32]. Neurons communicate through synapses to form complex neural networks. Studies have shown that 0.138 THz and 7 mW/cm^2^ irradiation can promote the dynamic growth of cortical neuronal processes. The slope and maximum amplitude of postsynaptic potentials in the hippocampal CA1 region increased after this treatment, indicating increased synaptic transmission efficiency [33]. Additionally, when IL brain slices were irradiated with 30–45 THz and 30 mW THz waves, the frequency, amplitude, and dynamic characteristics of excitatory and inhibitory postsynaptic currents were altered [34]. Calcium is a key signaling molecule, and changes in its concentration are essential for neuronal information transmission [23]. THz waves have been shown to increase both the neuronal Ca^2+^ concentration and Ca^2+^ current, increasing the number of neuronal Ca^2+^ events [34,35,36]. Several studies have suggested that THz waves may have a negative regulatory effect on neuronal function. For example, 34 THz waves at 166 mW/mm^2^ reduced the excitability of glutamatergic neurons in the orbitofrontal cortex of depression model mice, thereby alleviating depression-like symptoms and improving cognitive function [37]. In contrast, our study revealed that THz waves positively regulate neuronal function. Specifically, THz waves increased activity, excitability, network firing, synaptic transmission, synaptic plasticity and glutamatergic neuronal calcium activity in IL and vCA1 neurons. In addition, the current and ion transport function of AMPAR were enhanced. At the protein level, THz waves exposure increased neurotransmitter release, receptor density, and signaling, further confirming the positive activation effect on neuronal function. The primary effect of THz waves on synaptic transmission in the IL and vCA1 is the increase in excitability, with the IL showing increased excitatory synaptic transmission and the vCA1 exhibiting increased excitatory and inhibitory synaptic transmission. This difference might be attributed to variations in neuronal distribution and firing patterns across these brain regions. It is worth noting that after THz waves exposure, the synchronous firing of vCA1 neurons increased, whereas that of IL neurons remained largely unchanged. This discrepancy may be attributed to structural differences between hippocampal and prefrontal cortical networks, as well as variations in the balance of excitatory and inhibitory neuronal activity [38,39,40]. These factors collectively contribute to the differential responses of IL and vCA1 neurons to THz wave stimulation. Future research should further investigate the sensitivity of various brain regions to THz waves, integrating electrophysiological and molecular biological approaches to elucidate the precise mechanisms underlying THz wave effects in distinct neural circuits.

THz waves have diverse effects on animal behavior, including improvements in cognitive function, alleviation of neural damage, and modulation of behavior. For example, 34 THz, 6 W/cm^2^ waves effectively inhibited the excitability of glutamatergic neurons in the anterior cingulate cortex by reducing the number of hydrogen bonds between glutamate molecules and GluA2, alleviating pain and anxiety-like behaviors [41]. Other studies have shown that 2.52 THz, 50 mW/cm^2^ waves significantly increase the travel distance and speed of zebrafish larvae [42]. Furthermore, 0.167 THz exposure over 5 d alleviated behavioral abnormalities caused by insufficient exercise stress in male albino rats [43]. Additionally, 0.14 THz, 90 mW/cm^2^ waves restored motor function in the hind limbs of mice with spinal cord injuries, reduced inflammatory responses, and improved neurological function [44]. In our study, THz waves did not significantly impact motor ability, anxiety levels, or object recognition memory in mice. However, the discrimination index in the Y-maze novel arm test increased. Long-term THz waves exposure to the IL led to an increase in spontaneous alternation rates in the Y-maze, improving working memory. In contrast, THz waves exposure in the vCA1 did not significantly affect working memory, possibly due to the deeper location of the vCA1, which results in lower THz waves energy reaching the region, thus limiting its impact on neuronal function. Future research should focus on optimizing the THz fiber transmission system to enhance the focusing accuracy and transmission efficiency of THz waves while minimizing energy loss. These improvements would increase the effective energy delivered to the vCA1 region. We also compared the regulatory effects of optogenetic activation and THz waves on working memory. Our findings showed that THz radiation of the IL produces similar neuroregulatory effects as optogenetic activation of vCA1-IL glutamatergic projections and IL glutamatergic neurons. THz waves offer a noninvasive means of precise behavioral regulation without the need for exogenous gene introduction, representing a promising new approach for noninvasive neuroregulation in preclinical and therapeutic contexts.

Omics technologies, particularly genomics, epigenomics, and proteomics, are invaluable tools in neuroscience research [45,46]. These technologies offer new perspectives on brain function and disease mechanisms. In a previous study, our group used 0.141 THz irradiation of primary hippocampal neurons and identified differentially expressed genes, such as CaMKIIδ, through proteomics. CaMKIIδ has been shown to promote synaptic plasticity via the NF-κB pathway [47]. Other studies have utilized RNA-seq to explore the effects of THz waves on neuronal gene expression, contributing to mechanistic insights [27,48]. Most previous studies have employed single-omics approaches, which can provide a limited understanding of brain function or disease mechanisms. In contrast, multi-mile-mile approaches offer a more comprehensive view of the biological processes involved. For example, through joint ATAC-seq, Hi-C, and snRNA-seq analysis, we found that THz waves exposure reduced chromatin accessibility at genes such as Taf6 and Mt3, which negatively regulate neural function, ultimately leading to increased neuronal activity. At the level of the 3D chromatin structure, a new loop near the Tshz3 gene was observed. Tshz3 is involved in regulating glutamatergic synaptic transmission and LTP. At the gene level, an increase in the proportion of excitatory neurons was noted, along with the upregulation of genes such as Epha4, Epha7, and Hpca, which are involved in synaptic plasticity, neural development, and signal transduction.

In addition, S. Hameroff’s vibrational theory suggests that microtubules resonate at terahertz frequencies, making them potential targets for terahertz wave modulation. Given their role in synaptic plasticity, terahertz waves may influence neural activity by altering microtubule dynamics [49]. Future research should investigate this hypothesis to enhance our understanding of terahertz-induced neural regulation mechanisms.

**Figure 6:**
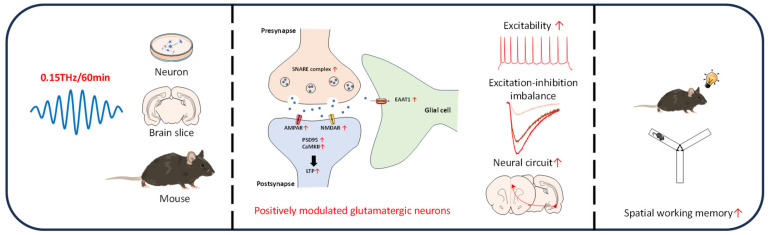
Schematic diagram of the neuromodulatory effect of THz waves.

In conclusion, this study revealed that THz waves specifically increased the activity of glutamatergic neurons. At the cellular, brain slice, and animal levels, THz waves increased the electrical activity and synaptic transmission, promoted the synaptic plasticity, and altered the neural circuit function of IL and vCA1 neurons. Molecular dynamics simulations found that THz waves enhanced the ion transport function of AMPAR. Western blot confirmed these changes at the protein level. At the behavioral level, THz waves noninvasively improved working memory, producing effects comparable to those of optogenetic regulation (Figure 6). Multiomics analysis revealed that THz waves regulated neural function by altering chromatin accessibility, 3D chromatin structure, and gene expression. As a noninvasive and precise neuroregulatory tool, THz waves hold significant potential for the treatment of neurological diseases and cognitive dysfunction. Future studies will further optimize THz waves parameters and explore their neural regulatory mechanisms, with an emphasis on ensuring safety for clinical applications.

## Data Availability

All data are available in the main text or the Supplementary Materials.

## Supplementary Materials

The following supporting information can be downloaded at: https://www.mdpi.com/article/10.3390/cells14050370/s1, Figure S1: Schematic diagram of in vitro and in vivo THz waves exposure; Figure S2: Temperature changes during 60 min of THz waves exposure; Figure S3: Effect of THz electric field on Na^+^ transmembrane; Figure S4: Effects of THz waves on the E–I balance of vCA1 neurons; Figure S5: Western blotting analysis of molecules related to glutamate metabolism after THz waves exposure; Figure S6: Effects of THz waves on the function of vCA1-IL neural circuits; Figure S7: Effects of THz waves on the activity of different subtypes of neurons in the IL; Figure S8: Comparative study of THz waves and optogenetic neuromodulation; Figure S9: Effects of THz waves on the three-dimensional structure of neuronal chromatin; Figure S10: snRNA-seq data QC.
