# Supplementary material for: THz Waves Improve Spatial Working Memory by Increasing the Activity of Glutamatergic Neurons in Mice
Source: Cells. 2025 Mar 3;14(5):370. doi: 10.3390/cells14050370 (PMC11898596; doi:10.3390/cells14050370)
Supplement: Supplementary file 1 [file cells-14-00370-s001.zip › cells-3497525-supplementary.pdf]

Supplementary Materials

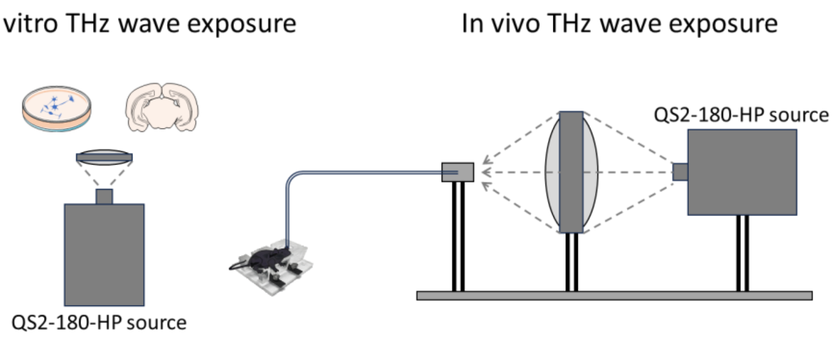

Figure S1. Schematic diagram of in vitro and in vivo THz waves exposure

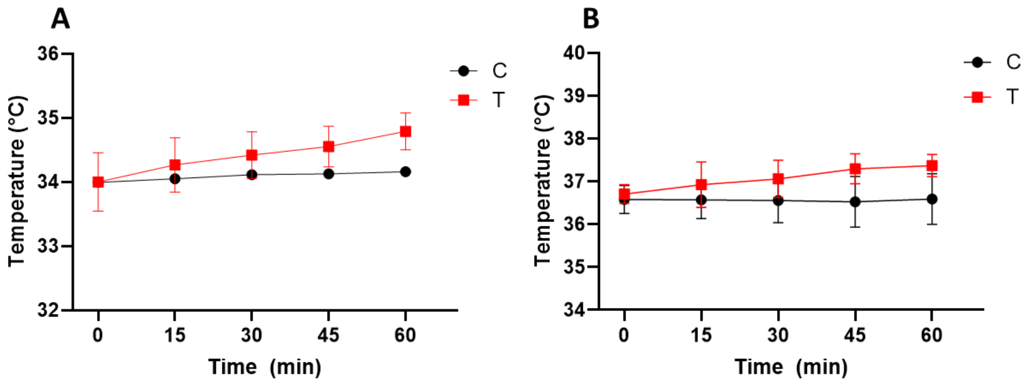

Figure S2. Temperature changes during 60 min of THz waves exposure

A: Temperature changes in neurons; B: Temperature changes on the mouse skull surface.

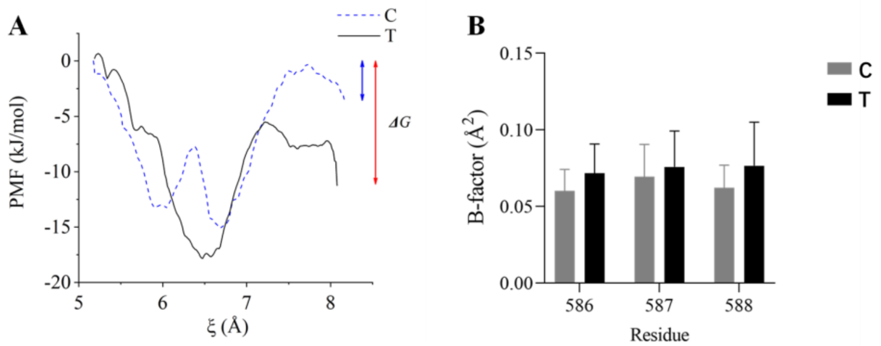

Figure S3 Effect of THz electric field on Na^+ transmembrane. A: PMF curve of Na^+ transmembrane through SF; B: B-factor statistical analysis.

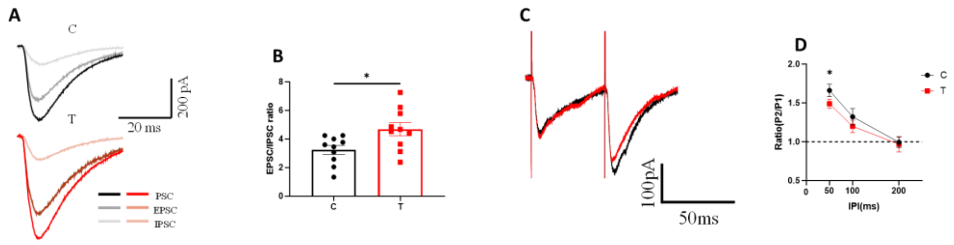

Figure S4. Effects of THz waves on the E–I balance of vCA1 neurons. A: PSC, EPSC, and IPSC traces of neurons; B: Statistical analysis of the EPSC/IPSC ratio; C: PPR of neurons; D: Statistical analysis of the PPR. * indicates $P < 0.05$.

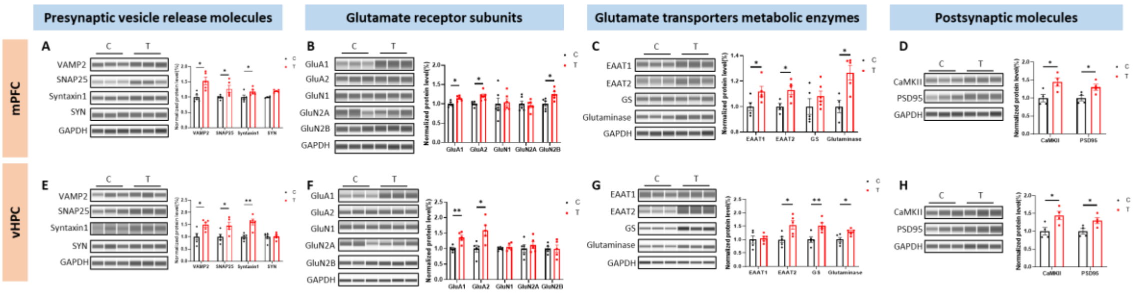

Figure S5. Western blotting analysis of molecules related to glutamate metabolism after THz waves exposure. A-D: Western blot analysis of vesicle release-related molecules, glutamate receptor subunits, glutamate transporter metabolic enzymes, and postsynaptic molecules in the IL; E-H: Western blot analysis of vesicle release-related molecules, glutamate receptor subunits, glutamate transporter metabolic enzymes, and postsynaptic molecules in the vCA1. * represents P < 0.05, ** represents P < 0.01.

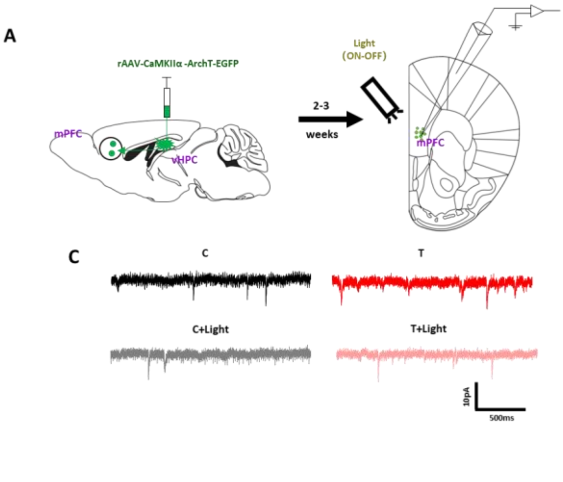

Figure S6. Effects of THz waves on the function of vCA1-IL neural circuits. A: Schematic diagram of virus injection and optogenetic experiments; B: Virus expression verification; C: IL neuron sEPSC; D: Statistical analysis of IL neuron sEPSC frequency. * represents P < 0.05.

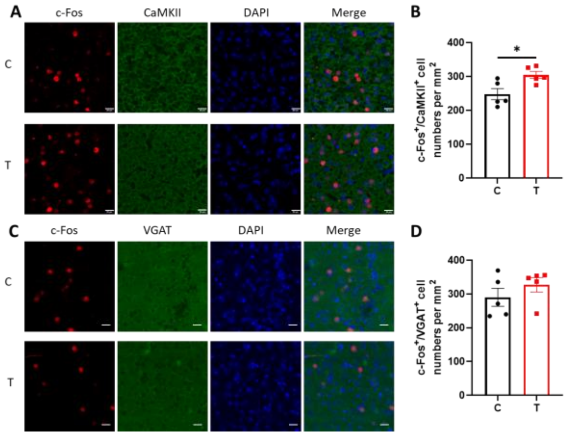

Figure S7. Effects of THz waves on the activity of different subtypes of neurons in the IL. A: c-Fos/CaMKII immunofluorescence costaining; B: Statistical analysis of c-Fos$^+$/CaMKII$^+$ cell numbers; C: c-Fos/VGAT immunofluorescence costaining; D: Statistical analysis of c-Fos$^+$/VGAT$^+$ cell numbers. * represents P < 0.05. Scale bar = 20 μm.

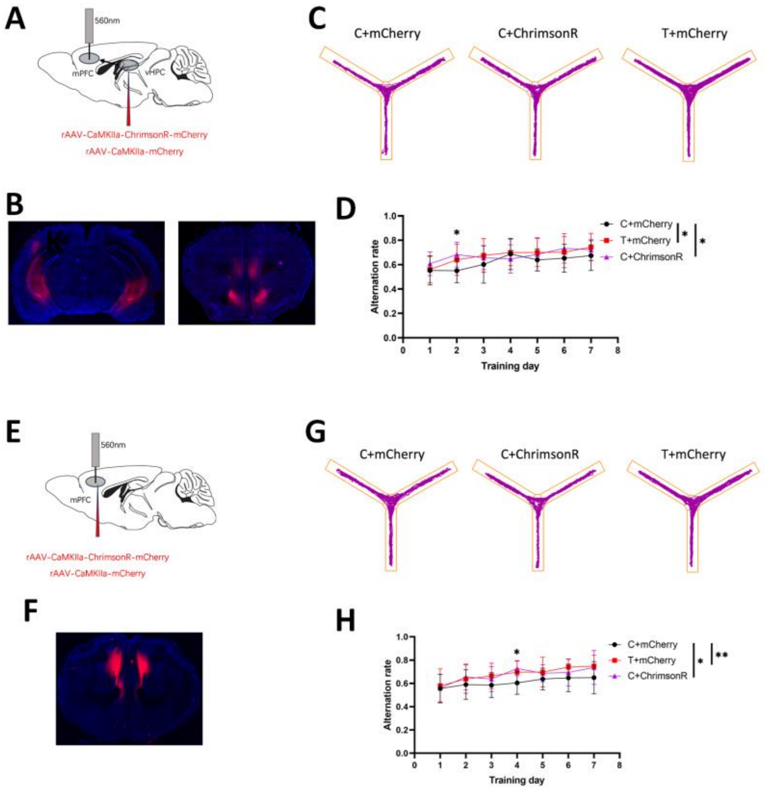

Figure S8. Comparative study of THz waves and optogenetic neuromodulation

A: Schematic diagram of virus injection for optogenetic activated vCA1-IL glutamatergic projections; B: Verification of the virus injection site; C: Trace diagram of the spontaneous alternation experiment in the mouse Y-maze; D Statistical analysis of the spontaneous alternation rate; E: Schematic diagram of virus injection for optogenetic activated IL glutamatergic neurons; F: Verification of the virus injection site; G: Trace diagram of the spontaneous alternation experiment in the mouse Y-maze; H: Statistical analysis of the spontaneous alternation rate. * represents $P < 0.05$, ** represents $P < 0.01$.

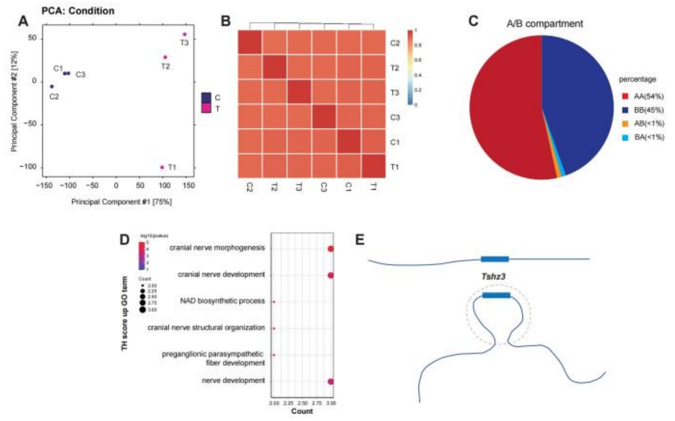

Figure S9. Effects of THz waves on the three-dimensional structure of neuronal chromatin

A. PCA plot using affinity data for all sites in the C and T groups. B. Correlation heatmap of Hi-C data in the C and T groups. C. Proportion of the A compartment/B compartment before and after THz radiation. D. Bubble plot of the enriched GO terms in the genes with differential TH scores in the T group. E. Pattern diagram of the gained loop after THz irradiation.

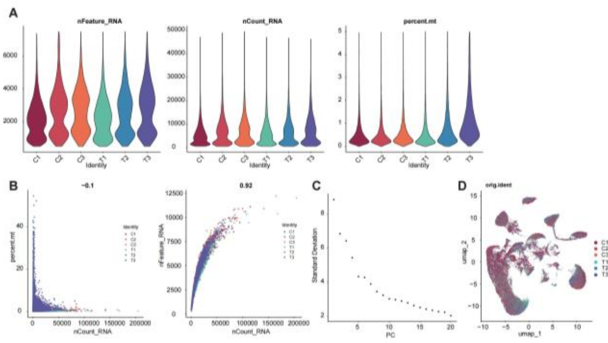

Figure S10. snRNA-seq data QC

A. Violin diagram showing the QC indices (nFeature_RNA, nCount_RNA, percent.mt) after data normalization, cell selection and filtration. B. Correlations of feature features (nCount_RNA-percent.mt, nCount_RNA-nFeature) are shown. C. Elbow plot showing the PC ranking according to the standard deviation to determine the number of clusters. D. snRNA-seq of brain tissue samples obtained from mice. UMAP clustering of single-cell transcriptomes colored according to sample.
